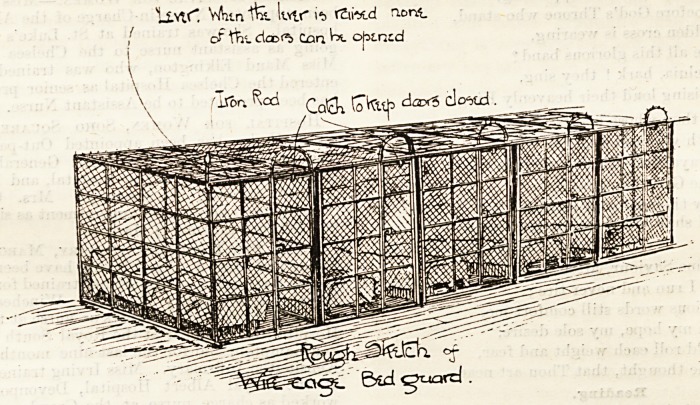# The Hospital Nursing Supplement

**Published:** 1896-10-31

**Authors:** 


"The Hospital" Nursing Mirror.

Being the Extra Nursing Supplement of "The Hospital."

[Contributions for this Supplement should be addressed to the Editor, The Hospital, 28 & 29, Southampton Street, Strand, London, W.C., and should have the word "Nursing" plainly written in left-hand top corner of the envelope.]

### News from the Nursing World.

### HOSPITAL FOR CONSUMPTION, BROMPTON.

The committee of the Brompton Consumption Hospital have purchased the freehold of ten additional houses in South Parade, at the back of the hospital, with the view of erecting thereon, in due course, a home for the nursing staff. The present accommodation is admittedly insufficient, and friends of the hospital will do well to forward with energy the speedy carrying out of the proposed extension. Recently Miss Davidson has instituted instruction in cookery, with demonstrations and lectures for her nurses, the present course being given by the Universal School of Cookery every Monday at half-past five p.m.

### CHELSEA HOSPITAL FOR WOMEN.

A considerable increase has just been made in the salaries accorded to the sisters, or charge nurses, at the Chelsea Hospital for Women, Fulham Road; £30 has hitherto been the limit of yearly payment, now the salaries are to be raised to £35 the second year, and to £40 for the third year. The salary of the night superintendent has been increased from £30 to £40 per annum. There are three charge nurses in the building, one over each floor. Miss Heather Bigg has just been able to promote one of her own staff to the charge of the Albany floor.

### NURSES ON TIP-TOE.

The development of flat-foot, resulting from long standing, often compels a would-be nurse to give up training, and many hospital nurses suffer with their feet, but the victims of this weakness will be amused at the reason assigned for it by a "hospital official" whose words we have recently seen quoted. Remarking that "nurses invariably develop a curious manner of walking after they have been in the wards for any length of time," he goes on to ascribe this to "the fact that when tending the sick they must make as little noise as possible. The chief sound in ordinary walking is caused by the heel, which touches the ground first. To avoid this many nurses walk on tip-toe. After a time great numbers of them cannot walk in the ordinary manner, and even when in the streets there is something springy and jerky in their method of walking."

### ASHTON SICK NURSING ASSOCIATION.

The Ashton and District Sick Nursing Association, like many a similar charity, finds its sphere of work increase with far greater rapidity than the subscription list, and the committee have just organised a fancy fair and sale of work, in the hope of obtaining some help thereby. Miss Bertha Mason, who is its treasurer and warm friend, made an excellent speech in opening the proceedings. Quoting some words of Lord Salisbury's on a similar occasion, in which he remarked upon the curious fact that often before men will support a religious organisation or an association which strives to alleviate the sufferings of the sick poor, they must have some "mundane attraction" in the shape of ball or garden party, Miss Mason said the committee had most reluctantly adopted such means of raising funds, and they now hoped it might bring them in not less than £100, and as much more as her audience should feel disposed to give. For twelve years the association had worked among the sick folk of Ashton, the cases having doubled and trebled, and the staff of nurses increased likewise, the only stationary department being the financial. An additional £250 per annum is needed to carry on the work satisfactorily.

### BAZAAR AT KETTERING.

Viscountess Hood, with her son and daughter, the Hon. Dorothy and the Hon. Neville Hood, was present on the second day of the bazaar held last week in the Victoria Hall, Kettering, in aid of the Kettering and District General Hospital. The first two days' sale produced excellent results, the total takings amounting to £1,800.

### CROYDON NURSES' INSTITUTE.

The report presented at the recent annual meeting of the Croydon Nurses' Institute was not an entirely satisfactory one, for it showed that while the private nursing branch was worked at a profit there was an adverse balance in the district nursing account. The vicar of Croydon, the Rev. H. Pereira, who presided, and the Mayor, who seconded the adoption of the report, strongly urged the need for increased annual subscriptions, and more help from the churches in the neighbourhood. Though the accounts of the private and district work are kept entirely distinct, the balance on the former is available to make up a deficit on the latter account, a step which both speakers rightly deprecated as "not a satisfactory way of going to work, for although it was true that both institutions were one, the committee would like to treat their nurses more liberally if they had the funds," and it was intended next year to open a reserve fund for nurses incapacitated from duty. We note from the report that the Croydon Institute is worked on the system of subscribers' letters, the rules being that "patients must obtain a subscriber's letter, which entitles them to the daily visits of a trained nurse for a month. After that time, if attendance is still necessary, a second letter must be procured. These letters are supplied . . . at the rate of four for a guinea."

### TOWCESTER DISTRICT NURSING ASSOCIATION.

Two meetings were held in Towcester Town Hall on October 21st in connection with the establishment of a district nursing association. Lady Hasketh took the chair in the afternoon, and was supported on the platform by representatives of all denominations, who cordially encouraged the new scheme. The Rev. W. H. Deane explained that the objects of the association were to engage a trained district nurse for the sick poor in their homes, to collect donations and subscriptions, to invite all ministers of religion to institute annual collections in their churches and chapels for the funds of the association, and to solicit and encourage the co-operation of all classes of the community in the work of the association. Several speakers testified to the urgent need for a competent nurse in the district. There was a large gathering at both afternoon and evening meetings, and a lecture on the advantages of trained nursing was given on each occasion by Miss Gethen. Mrs. Deane has been elected chairman of the association, Miss C. A. Phipps treasurer, and Mrs. Whitton secretary.

### MIDDLESBROUGH EXHIBITION.

An industrial exhibition has been organised in Middlesbrough by the Mayor, Councillor Phillips, for the benefit of three local charities—the North Riding Infirmary, the Cottage Hospital, and the Nurses' Home. The proceeds during the first week amounted to £1,000.

### THE GUARDIAN'S VIEW.

Guardians not infrequently wonder why nurses should be dissatisfied with their lot under the poor law; for surely they get enough to eat! At a certain workhouse in Lancashire, we are told that a nurse on night duty lately received for her week's ration six pounds of beef, two pounds of bacon, plenty of potatoes, and an ample supply of flour and currants and other cooking requisites, besides her tea and sugar. This was ample, nay, it was wastefully ample, but it was all uncooked! The next week was a pork week, and, as well as her two pounds of bacon, she had six pounds of pork served out to her, also raw. So it would appear that these precious Guardians, after screwing up their courage to the extent of paying for a nurse, force her to waste her time frizzling bacon and cooking steaks, puddings, and potatoes, instead of looking after her patients. The poor law and its administration is truly a wonderful thing, and full of marvels.

### A WOLF IN SHEEP'S CLOTHING.

A suggestion has been made in regard to the uniform of the female sanitary inspectors recently appointed by the vestry of St. George's, Southwark, which, we think, will hardly meet with the approval of those who appreciate honesty, and like things, and even people, to appear what they really are. It was proposed by a sub-committee of the vestry that this good lady should don the uniform of a nurse, apparently in order that she might the more easily penetrate the slums which she will have to visit in the course of her daily, and it may be added her nightly, work. Of course, as nurses, we protest against any such use of what has come to be looked on as the nurse's uniform. At the present time, unfortunately, this costume does not mean much. Still, any meaning it has, and any protection it may give, is the result of much honest nursing work, done amid much difficulty, by a long series of self-denying district nurses, and the assumption of it by a sanitary inspector, just because it is imagined that in such disguise her work will be more easily performed, is quite as much a false pretence as it would be if a debt collector or a process server were for the same reason to go about rigged out in the same costume. We doubt, however, whether any such mean device would give the hoped-for protection to these sanitary officials. There is a strong sense of honour among the denizens of the slums. They may not love a "copper," but they absolutely loathe a "'tec," and to the minds of these people an agent of the law rigged out in nurse's costume would be a detective all over. Such a "wolf in sheep's clothing" would be looked on as a spy, and would receive far less consideration than if she walked in openly in her official uniform. We are glad to hear that the proposal was referred back for further consideration.

### OPENING OF NEW HOME, CHELSEA INFIRMARY.

The new Nursing Home of the Chelsea Infirmary, just completed, was opened on Wednesday by the chairman of the Board of Guardians, Mr. Douglas Hamilton-Gordon. The opening ceremony took place at half-past three, and Mr. and Mrs. Gordon further celebrated the occasion by a reception at the Home in the evening. We gave a short description of the Home in "The Hospital" Nursing Mirror for October 3rd.

### M. FELIX FAURE AT RAMBOUILLET.

The President of the French Republic takes many opportunities of visiting hospitals and charitable institutions. While staying recently at the Chateau de Rambouillet he went to inspect the hospital, an institution founded last century by the Comtesse de Toulouse also sending presents of game for the patients, and giving a donation towards its funds. The hospital at Rambouillet is nursed by nuns, and to one of these, who had been working for forty years among the sick and infirm, M. Faure awarded a medal of honour for her long service.

### ST. HELENA HOME.

The St. Helena Home for Trained Nurses and Paying Patients, Grove End Road, N.W., has ceased to exist. The immediate reason for its dissolution is the acquirement of the property upon which the home stood by the Manchester, Sheffield, and Lincolnshire Railway Company, but we understand that there is no idea of making a fresh start, and that the institution has been definitely broken up. Miss Robertson and her staff of nurses have presented their library to the Royal British Nurses' Association for the use of its members.

### SHORT ITEMS.

Nurse Thwaites has been appointed Queen's Nurse at Loughton, Essex.—The Upton-on-Severn Board of Guardians have decided to subscribe one guinea annually to the Workhouse Nurses' Association. At Swaffham, in Norfolk, the guardians have passed a similar resolution.—Her Royal Highness the Duchess of Teck has promised to be present, early in the coming year, at the opening of a new wing now being added to the Children's and General Hospital at Walthamstow.—Princess Henry of Battenberg has sent a donation of ten guineas towards a fund which is being raised to build a cottage hospital at Cowes.—Miss Annie Anderson, M.D., has been appointed to the Honorary Assistant Physiciancy at the Clinical Hospital for Diseases of Women and Children, Manchester.—A successful concert was given in the Ulster Hall Annexe on behalf of the Belfast Hospital for Sick Children on October 16th.—Lectures are being given on "Home Nursing" at Selborne and Ropley, in Hampshire, by Miss Milwood Manners.—The annual demonstration of the friendly societies of Barry, of which the proceeds are always devoted to the Barry Nursing Association, suffered somewhat this year by reason of the very bad weather. Nevertheless, there was a good gathering, and we hope the results will be satisfactory for the Association.—The first annual meeting of the Corwen District Nursing Association was held last month. The report of the Inspector of the Queen's Jubilee Institute, with which the association is affiliated, expressed satisfaction with the work done during the year.—Lady Warwick presided the other day at the annual meeting of the Dunmow District Nursing Association, and earnestly appealed to her audience to help in providing good nursing for the sick poor in their own homes.—H.R.H. the Princess of Wales has sent a donation of £20, in aid of the Extension and Improvement Fund of Qeen Charlotte's Lying-in Hospital, Marylebone Road, of which she is a Vice-Patron.

### Hygiene: For Nurses.

By JOHN GLAISTER, M.D., F.F.P.S.G., D.P.H.Camb., Professor of Forensic Medicine and Public Health, St. Mungo's College, Glasgow, &c.

### XXX. — PREVENTABLE DISEASES — EPIDEMIC — ENDEMIC—PANDEMIC—THEIR PRIME CAUSES.

THE term "preventable" applied to diseases is one of comparatively modern application, just as the word "preventive" has been prefixed to medicine as a synonym of sanitary science. Preventive medicine deals with the prevention of disease *ab initio*, and of the spread of certain diseases by removing the conditions by which they may be generated, or propagated. In its practical application, it implies some measure of State control. The class of infective diseases, and, indeed, other general preventable diseases, have compelled the attention of the Legislature, which has passed a series of Acts of Parliament, from which has arisen a huge network of sanitary machinery. It may be said that, to some extent, all diseases may be prevented or avoided by proper care being exercised on the part of individuals. But this implies a knowledge which the average person does not ordinarily possess. However, errors of eating and drinking, objectionable physical habits, abuse of the body in any form, undue or careless exposure to chills, &c., are a few instances of causes of illness which are well within the power of individuals to avert. On the other hand, there are certain diseases, the causes of which are beyond ordinary control, but which may be controlled or regulated by proper regulations laid down by a controlling authority, and carried out faithfully by individuals. The object of such is the protection of the people *en masse*, and to frustrate the negligence of the careless and ignorant. To this category, therefore, belongs the preventable disea es, those due to combined overcrowding and filth, as typhus fever, or to overcrowding alone, as phthisis and other lung affections, or to insanitary conditions of the sewage-disposal system, as diphtheria and enteric fever, or to polluted water, or milk, and to like diseases from like causes. To this class also belongs those diseases which are communicable from animal to animal, and from animals to man. The principal characteristic of the specific preventable diseases is their communicability from man to man, and from animals to man, either by infection or contagion. This has been realised for ages, and long before their causes were known. Then, the causal factors were mysterious entities which were looked upon as inflictions of punishment by God for the sins of the people, the appropriate remedy for which were prayers and fastings. In the word influenza, we still retain evidence of the mystery of the cause of the disease — the influence which struck down the people. The last twenty-five years has done more to clear up the mystery regarding prime causes than the previous two centuries, and to-day we are able to lay our finger on the causes of several of the scourges of humanity.

Communicable diseases present themselves in one of three forms, viz., the epidemic, the endemic, and the pandemic. The word epidemic (Gr. *epi*, upon; *demos*, the people) literally means something that comes upon the people. An epidemic disease is, therefore, one which, independent originally of local causes, attacks a number of persons in a population within a short time. The cause belongs more to the people than to the place. The word endemic (Gr. *en*, among; *demos*, the people) means something that prevails among the people, usually, however, as isolated cases. It is dependent upon local causes, and belongs more to the place than the people. The word pandemic (Gr. *pan*, all; *demos*, the people) means something that attacks all the people of a country, or the peoples of all countries, about one time, and the infective material is conveyed very largely by atmospheric agency, and also follows trade routes. Influenza is a typical pandemic disease, and its recent widespread visitation of the nations of the earth demonstrates its pandemicity. The following are the principal preventable diseases, viz., small-pox, chicken-pox, scarlet fever, measles, rötheln or German measles, typhus, enteric, relapsing and yellow fevers, cholera, influenza, whooping-cough, diphtheria, erysipelas, puerperal fever, pneumonia, and mumps; and, with a limited infectivity, tuberculosis. These may be called the infective type. Glanders, anthrax (known as wool-sorters' disease and malignant pustule in man), foot and mouth disease, venereal diseases, Egyptian and other forms of ophthalmia, leprosy, and rabies may be taken as instances of the contagious type. There are other diseases which may be deemed preventable, but which are due either to mal-nutrition or local climatic conditions, such as rickets, scurvy, malaria, gout, and rheumatism.

Let us now consider the causal factors of infective and contagious diseases. From time to time these have given rise to a large amount of speculation, and various theories have been propounded to account for the phenomena which result from their malign action. The three principal theories are those with which the names of Bastian, Béchamp, and Pasteur are associated, and in that order they demonstrate the evolutionary stages towards truth. Bastian's view was that, when fermentation occurred in any liquid contained in a vessel from which air was excluded, the fermentative action was due to the chemical condition of the substances in the vessel, and not to anything that was introduced from without. The classical experiments of Tyndall and Pasteur, however, entirely demolished this idea. They conclusively proved that if an organic liquid was rendered sterile by boiling, and the vessel sealed against the entrance of air, that the liquid would keep "sweet" for an indefinite time, thereby showing that the cause of the fermentative change was not in the sterilised fluid, but in something that came from the air, viz., living micro-organisms. Béchamp thought that diseases of the fermentative type, *i.e.*, specific infective diseases, were the result of an organised and solid blastema (as he called it), microscopic in size, to which he gave the name microzyme (Gr. *mikros*, small; *zyme*, yeast); that these microzymes in the course of their development within the body generated a ferment which he called zymase, and that the two together formed protoplasm. He did not believe that infective diseases were caused by micro-parasites introduced into the body from without, but by the microzymes themselves, which, acting abnormally, produced a vitiated ferment, and became transformed into microbes. He further thought that these microbes should not be looked upon as foreign bodies, but entities existing normally in the body, but having abnormal action.

The doctrine of Pasteur and Tyndall, which came to be called the microbic theory, is, that the specific causes of these diseases are living micro-organisms, which, when introduced into the body, cause a series of phenomena to develop, the most prominent of which, due to the growth and multiplication of these organisms in the tissues or blood of the affected person, is fever. These organisms are, therefore, parasites, in respect that they live and thrive and multiply solely at the expense of their host.

Based upon these facts, Koch has formulated the following test rules, whereby an infective disease should be so designated, viz.: (1) A microbe must be found in the blood or tissues of the person or animal which is suffering, or has died from, the disease; (2) this microbe, taken from the body, must be artificially cultivated, in a suitable culture medium, out of the body, in a series of generations, with no possibility of any other microbe being introduced during the process; (3) the microbe, from one of these cultures, on being inoculated into the body of a healthy animal, ought to reproduce the same phenomena of disease, as those exhibited in the animal or person from which it was taken; and (4) the microbe must be proved to have multiplied in the body of the animal inoculated.

So far as our knowledge goes at present microbes breed true; that is to say, one does not become transformed into another form which has a totally different action. Inasmuch as after sowing turnip seed we can calculate only on a crop of turnips, and not carrots, so does the microbe of tubercle, or cholera, or leprosy, cause tubercular disease, or cholera, or leprosy, and is not metamorphosed to cause pneumonia, or enteric fever, or small-pox.

### Trained Nurses' Clinic.

XIV.—NURSING OF CHILDREN.—(*Continued.*)

Probably most nurses consider that obedience is one of the first things which they are entitled to demand from the sick whom it is their business to serve. In this respect children prove good patients, generally speaking, for they are accustomed, more or less, to do what they are told. In fact, it is often easier to secure compliance with the doctor's wishes from a wayward child than from the man who has attained to years of discretion.

In both cases the judicious trained nurse can usually secure obedience sooner or later, and by exercising tactful sympathy she may gain her end without friction. It is well to remember that even a young child has some justification for questioning the authority of a strange person into whose hand he has been transferred, often without any warning. Feeling ill, and unlike his normal self in every particular, the small child's irritability is susceptible of being increased if he finds himself, without explanation, cut off from familiar faces.

Time will reconcile him to the new-comer if only she has preliminary patience, and in the meanwhile he yields to her obedience which probably he would withhold from his mother and sisters. If the little patient be docile by nature the nurse's difficulties are inconsiderable, but she has experiences as variable as the dispositions of her successive cases. In return for his obedience, whether it be given willingly or grudgingly, the child has certain rights of his own. So long as he does what is necessary he is entitled to freedom from unnecessary restraints. All possible rules should be relaxed in his favour. The thoughts of a sick child should be encouraged to take pleasant directions; he should not be environed with things likely to have a depressing influence upon him.

Even the sick man or woman is disproportionately affected by trifles, of which each is oblivious in health, often shrinking, therefore, from mentioning those fancies to which ill-health has given rise. How much more does the feverish child abstain from complaints. He may require coaxing to swallow the beef-tea which he has petulantly pushed aside, and he will resent coercion in other matters of diet, whilst enduring in silence numerous small trials which could be easily removed.

Hence, to those who have made the requirements of sick children their special study, it appears evident that a consideration of their idiosyncrasies on the part of the nurse, is to the full as important as the unquestioning obedience required of the patients.

An ignorant or unsympathetic person is capable sometimes of rendering such attention to the sick as will help the latter to struggle back through convalescence to normal health. This holds good even with children, but such attentions are wholly inadequate. The feebleness of ill-health and the imperative needs of acute diseases demand for the patients such skilled attendants as shall make their restoration to health as little of a struggle as possible. The forestalled wish, the presence of hygienic surroundings, the absence of all that is detrimental to a perfect recovery—all come within the province of the trained nurse. If she is an adept in the art of nursing children, she has it in her power to reduce their sufferings, and to control their discomforts to an extent which probably only an experienced physician can properly estimate.

The terrors of imaginative children are so real that they must never be overlooked by those in charge of them, nor must they be lightly dismissed as nonsense. They are not cast out of the child's head by a sarcastic or humorous remark; but fear of ridicule will assuredly drive the poor victim of unsubstantial fancies to concealment of his sufferings. No young people, and not many older ones, can bear with equanimity "being laughed at." The process is destructive to their self esteem, and it also seems unfair to be jeered at for the unwelcome terrors which they have undergone.

The gentle encouragement given by the nurse in whom confidence is placed, is often instrumental in influencing the child's whole future.

Finding sympathy instead of contempt, he may confide to her by degrees a number of fancies which have influenced him before his present illness, and which would eventually render him a pitiably nervous little creature, save for his present confession and the consideration with which it is received.

Respect is the due of children, and so is courtesy, yet only the best men and women accord them either. It is pitiable to hear them blamed for ill-manners, greediness, and temper, for which the parents or guardians alone ought to be reproached, the undesirable characteristics being often transmitted by heredity and fostered by example.

Perhaps undue praise is less dangerous to the recipient's character than unjust blame, but it should be avoided in the case of sick children. If the adult in charge gives fulsome approval to every compliance with orders, the patient may grow to consider the praises monotonous, and he may be inclined to think the obedience superfluous which is acknowledged so elaborately. In dealing with children it is hardly wise to be content with the present moment: it is better to look ahead, for the young folks have long memories, and resent inconsequence in others.

The sick child should be treated consistently, and by this means his nurse influences him in the best kind of way. If he be indulged injudiciously one day, and refused similar privileges on a subsequent occasion, he will feel ill-used, and who can blame him? He does not reason, but, judging alone from facts, he sees the inconsistency of the grown-up person, and regards it with scant favour. He does not say, "Why cannot I have that again to-day?" but rather, "Why did you give it me yesterday, and refuse it to-day?"

An unspoilt child's sense of justice is a very fine feeling, and shame to those who wound it. It is, perhaps, the greatest evil which can be unconsciously inflicted on a child, and such wounds leave scars. "It isn't fair!" is the ejaculation which seems to come so easily from the little creature who feels, without being able to explain, that on him has fallen something evil beyond his desserts. His own helplessness and his powerlessness to explain his grievance will weigh heavily on his sensitive mind.

No injustice is perhaps more keenly felt than any attempt at deception in the sick room. A child may resent having to take his medicine, but he will be much more angry if he finds himself tricked into swallowing a nauseous dose under the impression that it was "something nice." He will never forget that he has been deceived; the lie, whether spoken or only acted, will impress itself on a mind keen to retain new impressions. Lies and deceitfulness in any form are forbidden to children, and are unfortunately sometimes punished with such severity as to ensure future attempts at concealment of similar follies. Yet it is seen that those superior and dogmatical grown-up people will themselves indulge on occasions in deceit with regard to obnoxious doses. If they also make promises which they cannot perform, simply "to pacify" the patient or to secure a cessation of his entreaties, the child is overpowered by the confliction of ideas. He feels that a lower standard is held sufficient for those who expect so much from him, requiring that he should live on a level of honour and truth far higher than their own. It may be want of thought on the part of the adult; but no child is excused for such a paltry reason, and in the nurse of whose knowledge and power he thinks so much, absolute truth and honour are expected.

### Nurses in 1896—Their Quarters, Hours, and Food.

[These articles exhibit the actual condition of affairs in the spring of the present year.]

### CHARING CROSS HOSPITAL.

### I.—Terms of Training.

Probationers are received for training at Charing Cross Hospital between the ages of 23 and 34. The rules state that the term of a probationer's training is a complete year, which may, however, be extended by the lady superintendent for another quarter; and each probationer is expected to pass such examinations as to her qualifications as may from time to time be prescribed. Entering the hospital on a month's trial, if considered suitable, probationers are required to sign an engagement to continue in the service of the hospital for at least two years longer than the term of probation, such agreement to be terminable only by consent of the council, should the reasons put forward appear to them sufficient. Probationers here, according to the usual custom, may be discharged at any time by the Lady Superintendent in case of misconduct, or should she consider them inefficient or negligent. The names of all nurses joining the hospital staff are entered upon a register, in which a record is kept of their conduct and qualifications. Certificates are granted on the satisfactory completion of their engagement, which certificate may be extended for any further period of service, and may also be cancelled in case of misconduct or inefficiency at any subsequent time. It should be noted that the nursing regulations at Charing Cross honestly state what is the actual fact at every hospital, that nurses are "on probation" for one year, continuing "in the service" for the remainder of their engagement. At some hospitals the probationary period is said to last for four years, but the work done by nurses in their second, third, and fourth years is in practice the same as that required of staff nurses elsewhere. The difference lies in the wording alone.

At Charing Cross ordinary probationers, *i.e.*, those who sign for a three years' term of service, are unpaid for the first twelve months.

Special or paying probationers are admitted for twelve months' training, for which the fee is fifty-two guineas, half this sum to be paid in advance. They enter upon a month's trial, and if accepted at its expiration are required to sign an agreement to complete the term. If a special probationer leaves at the end of the first month a proportionate amount of the fee is returned to her. The names of special probationers are entered upon a register as in the case of the ordinary probationers, and they are accorded a certificate by the Council on the satisfactory completion of their engagement, and on the recommendation of the lady superintendent. The age considered desirable is from twenty-three to thirty-five. Training and rules are the same in the case of ordinary and special probationers. Special probationers, if suitable, are allowed to continue service on the paid staff, and may become eligible for appointment as sisters of wards.

### II.—Hours of Work and Times Off Duty.

Nurses and probationers go on duty in the wards at seven a.m., the former leaving them in the evening at half-past eight, the latter at eight p.m., and probationers take it in turn to return one to each ward at half-past eight to see if anything has been forgotten, remaining only for a short time. Nurses are daily off duty for two or three hours, ten a.m. to twelve noon, two to five p.m., or six to eight p.m., with half an hour each for dinner and tea. Sisters go on duty in the morning at eight a.m., leaving their wards for supper in the evening at a quarter past nine. They are off duty from two to five p.m., or six to twenty minutes past eight. Besides these hours, probationers have three hours off on Sunday, sisters and staff nurses have leave of absence on Sunday afternoons and mornings alternately, one Sunday from ten a.m. to one p.m., and the following week from half-past one p.m. for the rest of the day. Night nurses are on duty from nine p.m. to half-past eight a.m., and are off duty for two and a half hours each morning. Night and day duty is taken in alternate three months; there are no permanent night nurses appointed. One day off duty is given to all the staff each month, and on these days nurses do not enter the wards at all.

Probationers have a fortnight's holiday at the end of their first six months and a month at the end of the year; sisters and nurses have a month each year. Miss Gordon also gives occasional leave now and again as she finds it possible and desirable, holding it better to grant such holidays to any nurse who may be the least unfit for duty than to risk a possible breakdown. The nurses are encouraged to go out as much as possible.

### III.—Meals.

Day nurses and probationers breakfast at half-past six, dinners begin at a quarter to one p.m., tea is served at half-past four p.m., supper for the probationers at eight p.m., and for the staff nurses at half-past eight. Sisters breakfast at half-past seven, dinner is at a quarter past one, tea at half-past five, and supper at a quarter past nine. All meals are served in the dining-room, except the early lunch and the night meals, which are taken in the ward kitchen. Night nurses have their breakfast at half-past eight p.m. and their dinner at half-past nine a.m. The dietary is similar to that at most hospitals, and all the staff are provided with "a sufficient allowance of beer or porter to take at meal times."

### IV.—Salaries and Uniforms.

As already mentioned, ordinary probationers are unpaid for their year of probation. They are supplied with board, lodging, washing, uniform, and medical attendance; for the second year they are paid £15, and for the third year £20. These wages are paid monthly, and the regulations provide that the sum of 5s. will be deducted out of each of the second twelve months, making together £3, to be retained as "caution money" until the expiration of the first three years. Salaries for staff nurses rise to £25 a year, and sisters' salaries are £30, the night sister's £40. Outdoor and indoor uniform is provided by the hospital, and washing expenses are also defrayed in the case of the regular staff. Special probationers pay for their own washing. The uniform provided consists of three dresses, twelve aprons, four caps, and one bonnet each year, with two cloaks in three years.

### V.—Nurses' Quarters.

The nursing staff at Charing Cross are accommodated partly in cubicles on the top floor of the hospital proper, partly in three adjoining houses in King William Street, just at the side of the hospital building, and connected with the wards on each floor. The cubicles are as comfortable as cubicles ever can be, though they are very small. Still each is provided with a whole or a half window, so that they are fairly light. A few of the probationers sleep two in a room. Nurses do not change their rooms when going on night duty. A certain number of cubicles at each end of the dormitories are shut off by a door, and their occupants all change over from night to day duty at the same time, thus keeping the quarters of the night staff separate, but avoiding a removal from one cubicle to another.

The sisters at Charing Cross do not have rooms off their wards. They are provided with comfortable bed-sitting-rooms in the home. One floor of the home is devoted to sisters, and one to special probationers, who have separate rooms. Here also are the general sitting-room and dining-room, the latter being far too small for its purposes. Charing Cross Hospital possesses plenty of site for the erection of a proper nursing home, and awaits funds to pull down the present old houses and make this needed improvement.

### Royal British Nurses' Association.

Special Council Meeting.

In view of the objection raised by Dr. Bedford Fenwick at the meeting of the General Council of the Association last week to the re-election of the honorary officers on the ground that they had not been specially nominated previously by the Executive Committee, a special meeting was called on Friday, the 23rd inst., in order to place the matter beyond dispute. It was explained that this was done merely as a matter of protection, legal opinion, while describing the bye-law on the point as "ambiguous," clearly stating that this precautionary measure must not be taken as an admission that the former election was not in order.

The chair was taken by Mr. Pearce Gould. After explaining the reasons which had led to the summoning of the meeting, Mr. Pearce Gould read two letters protesting against the re-election of Sir James Crichton-Browne as vice-chairman, one from Miss Homersham and the other from Mrs. Okell, of Bridgewater Infirmary. In accordance with a resolution passed at a meeting of the Executive Committee that afternoon, Mr. Gould then formally proposed the re-election of the honorary officers *en bloc*, Sir James Crichton-Browne, Mr. Pickering Pick, and Miss Thorold as vice-chairmen, Mr. Langton as hon. treasurer, Mr. Fardon as medical hon. secretary, and Mrs. Dacre Craven as nurse honorary secretary, Miss De Pledge seconding the resolution.

Miss Burr protested against Sir James Crichton-Browne as a "prejudiced chairman," and Dr. Bedford Fenwick congratulated the Council on "setting this matter right," reiterating his grounds for considering the election of last week irregular. Dr. Gant and another member of the Council spoke of the ability and tact with which they felt Sir James Crichton-Browne had carried out his duties in the chair, duties in the discharge of which he had shown so much wisdom and forbearance; expressions evidently shared by the Council in general, for they met with warm applause.

At the insistence of Dr. Bedford Fenwick the names of the honorary officers were put up singly for election, with the result that out of some seventy members present four votes were recorded against Sir James Crichton-Browne, two each against Mr. Pickering Pick and Miss Thorold, one against Mrs. Dacre Craven, while Mr. Langton and Mr. Fardon were re-elected *nem. con.*

In regard to the report of Sir James Crichton-Browne's speech, which appeared in our issue of last week, he desires us to say that "the statement he attributed to Dr. Bedford Fenwick, and asked him to justify or explain, that the Association was £800 in debt, was promptly repudiated by that gentleman, upon which he (Sir James) immediately withdrew it, and expressed his regret for having misunderstood Dr. Bedford Fenwick, adding that the treasurer estimated the liabilities at about £400."

### Death in Our Ranks.

Miss Rachel Sutton died at Eastbourne on Saturday, October 24th, of typhoid fever, contracted from a patient whilst in discharge of her duties. Miss Sutton received her training at the Victoria Hospital, Lewes, and has been on the staff of the Borough Sanatorium, Eastbourne, since last November. Her early death is deeply mourned by the nurses with whom she has at any time worked, as her unvarying good temper and cheerful willingness to sacrifice her own wishes for the benefit of others has endeared her to all.

We regret to announce the death of Miss Annie Pinfold, nurse probationer at Guy's Hospital, which took place at the hospital on October 13th from typhoid fever. Nurse Pinfold had worked at the London Fever Hospital for three years before entering Guy's early this year. The funeral was on the 16th instant at Woking, the first part of the service—at which some sixty members of the nursing staff were present—being previously conducted in the hospital chapel.

### Our American Letter.

Miss Clara Barton, President of the American Red Cross Society, whose expedition to Constantinople for the purpose of distributing relief to the Armenians excited much interest in America, has now returned to New York. Miss Barton states that no difficulty was thrown in the way of herself and her party, the Sultan and his advisers apparently accepting their mission as one of mercy only, and everything was done to assist them in carrying it out.

A change has just taken place in the matronship of the Rufus S. Frost General Hospital, Chelsea, Mass., Miss Margaret M. Robertson, who has presided over the nursing department for six years past, being succeeded in that post by Miss Florence F. Rice. The trustees of the hospital met to bid farewell to Miss Robertson and welcome her successor, and adopted resolutions warmly thanking the former lady for "the very able manner in which she has performed her duties as matron for the past six years," and wishing her every success in the new field of service upon which she was about to enter. Miss Rice, who has taken up Miss Robertson's work, is a graduate of the Massachusetts General Hospital, and has successively held the posts of Superintendent of the Lawrence City Hospital and at the Memorial Hospital, Worcester. Miss Rice also spent some time in Italy nursing at St. Paul's House, Rome.

Miss Frances M. Quaife has been appointed Superintendent of the Hospital and Training School for Nurses at Touro Infirmary, New Orleans, La. Miss Quaife graduated at the New York Hospital Training School for Nurses, and has held the position of Nurse-in-Charge at the Montreal General Hospital.

The nurses at Brooklyn and Long Island lately held the first quarterly meeting of their Associated Alumnæ and Central Registry, at which Miss Merritt, the President, gave an excellent address, telling her audience of the recent Convention at Manhattan. This action of the Brooklyn nurses is interesting, because it is believed to be the first instance of an associated alumnæ in any United States city, and its progress will be eagerly watched by nurses. The registry, too, is the first of the kind under such management. Miss Lightbourne, a Brooklyn nurse, has been chosen as registry superintendent.

A new hospital has just been completed at Proctor, built and fitted up by the Vermont Marble Company especially for the benefit of the company's employés and their families, though open to others as well. With the view of interesting the people of the village in their hospital, a board of managers drawn from the inhabitants has been formed, into whos hands funds for its support will be given from time to time by the company. The hospital is to be practically a free institution.

Among other hospital additions and alterations should be mentioned the new private rooms just added to the Paterson General Hospital, seven in number, and all furnished by private subscription, and fitted in a thoroughly up-to-date manner. The Nurses' Home is fitted up in the old part of the building on Market Street. A new ward for the treatment of consumptive patients has been erected at the Orange Memorial Hospital through the kindness of Mr. F. M. Shepard, who gave the money for the building in memory of his son. Mr. Shepard's children have combined to defray the cost of the furnishing of the new wing.

### Where to Go.

Royal British Nurses' Association.—The Secretary requests us to state that the first of the course of demonstrations on "Invalid Cookery" will be given by Miss Earle, Diplomée of the National Training School of Cookery, at 17, Old Cavendish Street, W., on Tuesday, November 3rd, at half-past two p.m. Admission at the door for single lecture —Members, 1s.; non-members, 1s. 6d. Tickets and syllabus for the complete course may be obtained from the offices of the Association.

### Everybody's Opinion.

[Correspondence on all subjects is invited, but we cannot in any way be responsible for the opinions expressed by our correspondents. No communication can be entertained if the name and address of the correspondent is not given, or unless one side of the paper only be written on.]

### ORPHANS CAGED.

The Medical Officer of St. Mary's Home, Broadstairs, writes: Pray do not waste your "regrets" on me and on my opinions as to the use of wire cubicles—I beg pardon, I should have said "cages"—in orphanages. I have expressed no views of my own as to whether or not they might be a source of danger in the event of fire. It is not a medical question, and my opinion is worth no more than that of any ordinary individual. These "regrets" and the other trivialities of your footnote, which I need not notice, may be a useful means of diverting attention from the main issue. But it is better to stick to the point. The point is that your "Medical Correspondent" has made an utterly unjustifiable and indefensible attack upon the Kilburn Sisterhood in asserting that the children under their charge are shut up during the night in wire cages strongly resembling those that are in use for wild beasts in travelling menageries. This peculiarly stupid slander was inserted, to my surprise (and may I say regret?), in the columns of The Hospital, and I have taken my share in repudiating and refuting it. Your correspondent's attack is not directed against me, but it clearly affects me and others who are connected with the institution. Do you suppose that the clergy of this parish would consent to minister to the inmates of St. Mary's Orphanage if abominations existed such as your "Medical Correspondent" asserts to exist—abominations which would be apparent to anybody? And do you suppose that the visitors and sympathisers would continue their support under such circumstances? And why should it be supposed that a practitioner of thirty years' standing, twenty-seven of which have been passed in Broadstairs, would tolerate such a state of things among his patients? Under such conditions I would not hold my appointment for an hour. The Sisters of the Church have suffered severely from these stale, old slanders. They are practically defenceless, for they see nothing of the literature of the Press, and to retaliate by going to law would, I am sure, be against their principles. Therefore, if criticism is necessary, it surely should be honest and temperate, otherwise it hardly comes up to the standard of chivalry. I am not their apologist. In some matters I differ strongly with them; but they have as good a right to fair play as any other member of society.

[We are pleased to find that Mr. Raven, Medical Officer at St. Mary's Home, admits that if things are as they were asserted to be in the letter of a "Medical Correspondent" they would be "abominations," and also that under such circumstances he would not tolerate such a state of affairs, and "would not hold his appointment for an hour." The issue is thus narrowed. We, therefore, reprint the portion of the letter of a "Medical Correspondent" in which the "cages" are described, and also repeat the illustration of the similar "cages" which exist at Kilburn, and ask Mr. Raven to be good enough to point out any error so gross as to absolve these cages from the charge of being "abominations," which he admits they would deserve if the description given conveys a correct idea of them. He may say that *all* the orphans are not confined in such cages; but that is beside the mark. If any of the cages in which the orphans at Broadstairs are confined are described with substantial accuracy in the letter we quote below, we think he is bound, in accordance with his letter, to resign his appointment in connection with an institution where such "abominations" exist.—Ed. The Hospital.]

Extract from Letter of a "Medical Correspondent," which appeared in "The Hospital," Oct. 3, 1896.

"In strange contrast to the noble proportions of the dormitories and their uninterrupted outlook upon the expanse of ocean, the poor infants who sleep therein are 'cribbed, cabined, and confined,' each within its own enclosure of iron bars, from which no escape is possible till the clips which fasten the prison doors are withdrawn. It is terrible to contemplate the consequences in case of an outbreak of fire under such circumstances; no escape would be possible for the unhappy inmates of the cubicles until the doors had one by one been unfastened. Attention has already been called by The Hospital to this matter in connection with the parent institution at Kilburn; and we understand that some arrangement for the simultaneous opening of the iron doors has been adopted there. At Broadstairs, however, each cubicle must be opened singly, and how this would be done in a moment of panic it is difficult to see. It is true that the older inmates are trained in the use of fire-extinguishing apparatus, and that some would probably be charged with the duty of releasing the children. Theoretical preparation and practical accomplishment are, however, two different things; and the question which the good Sisters should consider is whether the economy of personal vigilance gained by the adoption of these cages is not dearly purchased when it entails the risk of a holocaust of innocents."

### Appointments.

### MATRONS.

The International Hospital, Naples.—Miss Mary E. Moore has been selected to fill the position of Matron at this hospital. Miss Moore was trained at St. Mary's Hospital, Paddington, afterwards holding appointments as staff nurse at the West London Hospital, at the Meath Home for Epileptics, Godalming, and at the General Infirmary, Sheffield.

Oldham Nursing Association.—Miss Mary T. Nicholson has been appointed Lady Superintendent of this institution. She received her training at the Thompson Memorial Home for Incurables, Lisburn, near Belfast, and at Oldham Infirmary, where she has worked for nine years as probationer, staff nurse, and the last four and a half year as sister of the women's and children's wards.

### For Reading to the Sick.

"DIVINE COMFORT."

**Motto.**

"So will I comfort you."—*Isa.* lxvi. 13.

**Verses.**

With silence only as their benediction,
God's angels come,
Where, in the shadow of a great affliction,
The soul sits dumb!—*J. G. Whittier.*

Though from the shadow of Thy peace
My feet would often stray,
Thy mercy follows all my steps,
And will not turn away;
Yea, Thou wilt comfort me at last,
As none beside Thee may.

O, there is nothing in the world
To weigh against Thy will!
E'en the dark times I dread the most
Thy covenant fulfil;
And when the pleasant morning dawns
I find Thee with me still.
—*Hymns and Meditations.*

Who are these like stars appearing,
These, before God's Throne who stand,
Each a golden cross is wearing,
Who are all this glorious band?
Alleluia, hark! they sing,
Praising loud their heavenly King.

These are they whose hearts were riven,
Sore with woe and anguish tried,
Who in prayer full oft have striven
With the God they glorified;
Now their painful conflict o'er,
God shall bid them weep no more.
—*Hymns Ancient and Modern.*

Oh, draw me Saviour, after Thee,
So shall I run and never tire;
With gracious words still comfort me,
Be Thou my hope, my sole desire,
On Thee I'd roll each weight and fear,
Calm in the thought, that Thou art near.

**Reading.**

"For the Lord shall comfort Zion; He shall comfort all her waste places; and He will make her wilderness like Eden, and her desert like the garden of the Lord; joy and gladness shall be found therein, thanksgiving and the voice of melody."—*Isa.* li. 3.

"God shall wipe away all tears from their eyes; and there shall be no more death, neither sorrow, nor crying, neither shall there be any more pain."

See the lovingness of the action. He has to send us much now that brings tears. He will wipe all tears away in that day. A tender mother sometimes must needs make her little child cry, for the child's good; but when the need is over, the good accomplished, how she takes up the grieved child in her arms, and kisses and wipes away the tears!

"He will swallow up death in victory, and the Lord God will wipe away tears from all faces."

"These are they which came out of great tribulation. . . . God shall wipe away all tears from their eyes."

An old promise, given long ago, and renewed in the last chapter of the Bible. God is waiting patiently for that day; and He expects us to wait patiently, too. Though His heart of Fatherly love is longing to set us free, He will not act too soon. Each burning drop that falls from the aching heart has its work to do. But soon the time will come, and then—

There shall be no more sorrow.
There shall be no more crying.

### Minor Appointments.

Ancoats Hospital, Manchester.—Miss Annie Oliver has been appointed Sister of the accident wards at Ancoats Hospital in place of Miss Hilton, who has recently resigned her position in order to take up work abroad.

West Ham Hospital, Statford, E.—Miss Clara Amoore has been appointed to the post of Sister at the Plaistow Fever Hospital. She received her training at the West Ham Hospital, where she gained an excellent certificate, and was promoted to the charge of the out-patient department.

The Hospital of St. Cross.—Miss Marion Norton has been appointed Charge Nurse at this hospital. She was trained at the London Temperance and at the Boston Hospital, and has worked at Chesterfield Hospital and Dispensary as charge nurse, and as special nurse at the Jessop Hospital, Sheffield.

Macclesfield General Hospital.—Miss Annie Hargreaves has been appointed Sister of the Children's Ward at the Macclesfield Hospital. She received her training at the Bolton General Hospital, and has since held the appointment of charge nurse at the Eston Cottage Hospital, Middlesbrough.

Chelsea Hospital for Women.—Miss Rosa Rolls has been appointed Nurse-in-Charge of the Albany floor at this hospital. She was trained at St. Luke's Hospital, Halifax, going as assistant nurse to the Chelsea Hospital in 1895. Miss Maud Elkington, who was trained at Ipswich, and entered the Chelsea Hospital as senior probationer in 1895, has been promoted to be Assistant Nurse.

Hospital for Women, Soho Square, W.—Mrs. Ethel Carew-Hodge has been appointed Out-patient Sister. She was trained at the West Kent General Hospital, and at Queen Charlotte's Lying-in Hospital, and has also had three months' training in massage. Mrs. Carew-Hodge has recently been holding an appointment as sister at the Royal Sea Bathing Infirmary, Margate.

Royal Sea Bathing Infirmary, Margate.—Miss Louise Fisher and Miss A. Diana Irving have been appointed Sisters at this institution. Miss Fisher trained for three years at the Royal Hants County Hospital, Winchester, having since worked for nearly three years as nurse at the Dorset County Hospital, for one year at the Royal South Hants Infirmary, Southampton, and for the last nine months as sister at the Kensington Infirmary. Miss Irving trained for three years at the Royal Albert Hospital, Devonport, and has since worked as charge nurse at the Croydon General Hospital, night sister at Town's Hospital, Glasgow, charge nurse of the Rawson Convalescent Home, Harrogate, and for the past ten months as sister at the Kensington Infirmary.

### Notes and Queries.

*Trained Children's Nurses.*

(35) Can you give me the address of the training home in London for children's nurses?—*St. Peter's, Bedford.*

You probably mean the Norland Institute, where ladies are trained to be children's nurses. The address is 29, Holland Park Avenue, W.

*Daily Nursing and Dispensing.*

(36) Will you kindly tell me where I can get information about the working of daily visits to paying patients by nurses? Also I shall be grateful for advice as to the best way to learn dispensing in a country town. Could I apply to a local chemist?—*Nurse Marah.*

Write to Miss C. J. Wood, Nurses' Hostel, 27, Percy Street, Tottenham Court Road, who has had experience in arranging "visiting nursing." With regard to your question as to dispensing, you should read the chapter on this subject in Burdett's "Hospital Annual" for 1894, where you will find full advice and lists of books recommended for study. You should certainly be able to arrange with a local chemist for lessons in practical pharmacy. Have you ascertained if private lessons would be obtainable at the County and Borough Infirmary?

*St. John Ambulance Classes.*

(37) Will you kindly tell me where these classes are held in London?

Write for information to the St. John Ambulance Association, St. John's Gate, Clerkenwell.

*Massage.*

(38) Can you tell me the address of Dr. Herbert Little, the Professor and Instructor of Massage?

We suppose you mean Dr. Fletcher Little, whose address is 32, Harley Street.

## Figures and Tables

**Figure f1:**